# Renal cell oncocytoma tumor evolutions: a case report

**DOI:** 10.3389/fonc.2024.1282508

**Published:** 2024-03-01

**Authors:** Alaa Khalid Alduraibi, Noora Bin Essa, Jamshed Bomanji

**Affiliations:** ^1^ Department of Radiology, College of Medicine, Qassim University, Buraidah, Saudi Arabia; ^2^ Nuclear Medicine Department, Kuwait Cancer Control Center, Ministry of Health Kuwait, Kuwait, Kuwait; ^3^ Institute of Nuclear Medicine, University College London Hospitals National Health Service Trust, London, United Kingdom

**Keywords:** renal cell oncocytoma, FDG PET/CT, I123 scan, thyroid cancer, renal cancer

## Abstract

This report delineates the clinical progression of a renal oncocytoma in a 38-year-old female, initially asymptomatic, over a three-year follow-up period following her treatment for papillary thyroid cancer. The timeline of this case is as follows: In 2016, the patient was treated with total thyroidectomy and I131 for thyroid cancer. During an annual follow-up, an incidental renal mass was detected via FDG PET/CT, initially characterized as a benign, non-FDG-avid renal oncocytoma. Over two years, this lesion demonstrated a remarkable increase in FDG uptake and a slight growth in size, coupled with new I131 uptake in subsequent scans. These findings led to a reassessment of the diagnosis, initially suggesting a potential small renal cell carcinoma (RCC). Histopathological analysis eventually confirmed the diagnosis of oncocytoma. This case is notable for the tumor’s unusual metabolic evolution and the challenges it posed in differential diagnosis.

## Case presentation

A 38-year-old woman with a history of papillary thyroid cancer (treated by total thyroidectomy and I131 in 2016, stimulated TG was 1.8). During annual follow-up, the patient had an elevated thyroglobulin (TG) with a negative I131 whole-body scan, for which she underwent further evaluation by FDG PET/CT, where she has shoulder and mediastinal metastasis.

**Figure 1:**
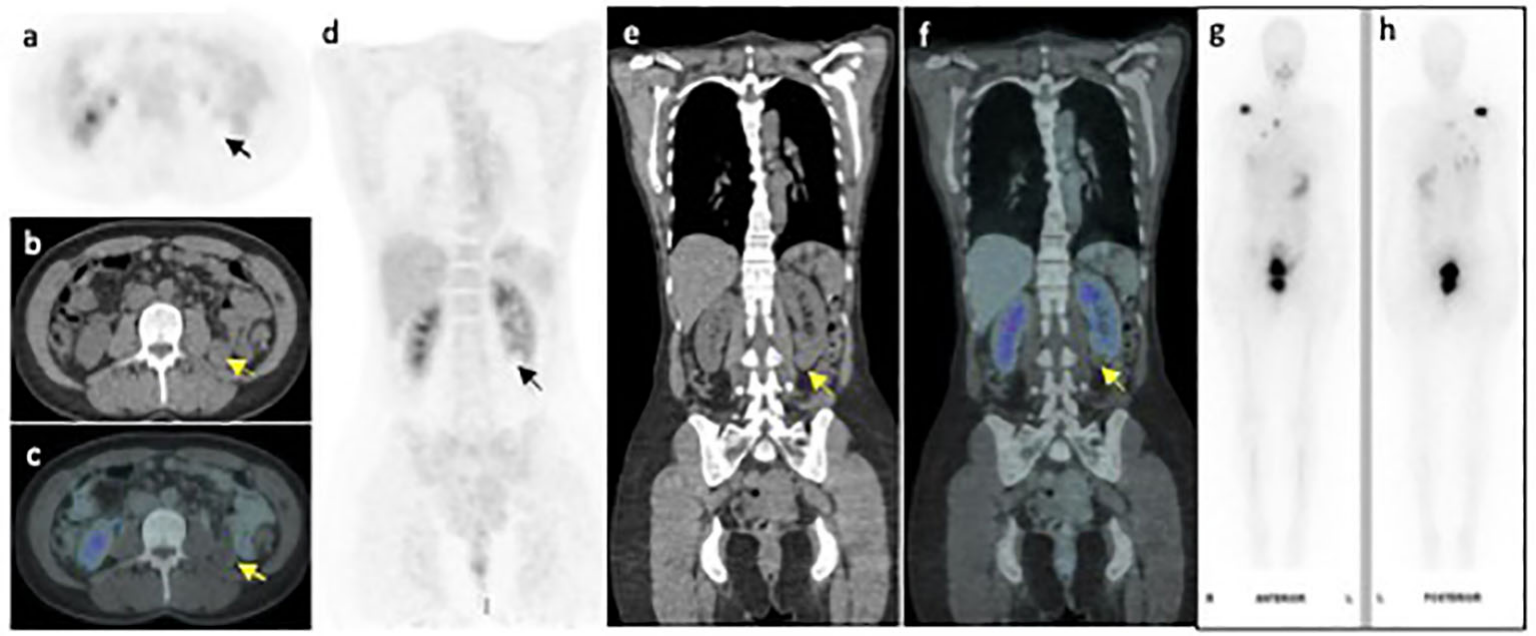
FDG PET/CT axial **(A-C)** and coronal **(D-F)** PET, CT and fused PET/CT images of the left lower pole renal mass (arrow) demonstrating no FDG uptake. Whole body I^131^ post therapy scan **(G, H)** performed within 6 weeks of the FDG-PET/CT scan, shows no appreciable I^131^ accumulation within the renal mass.

**Figure 2:**
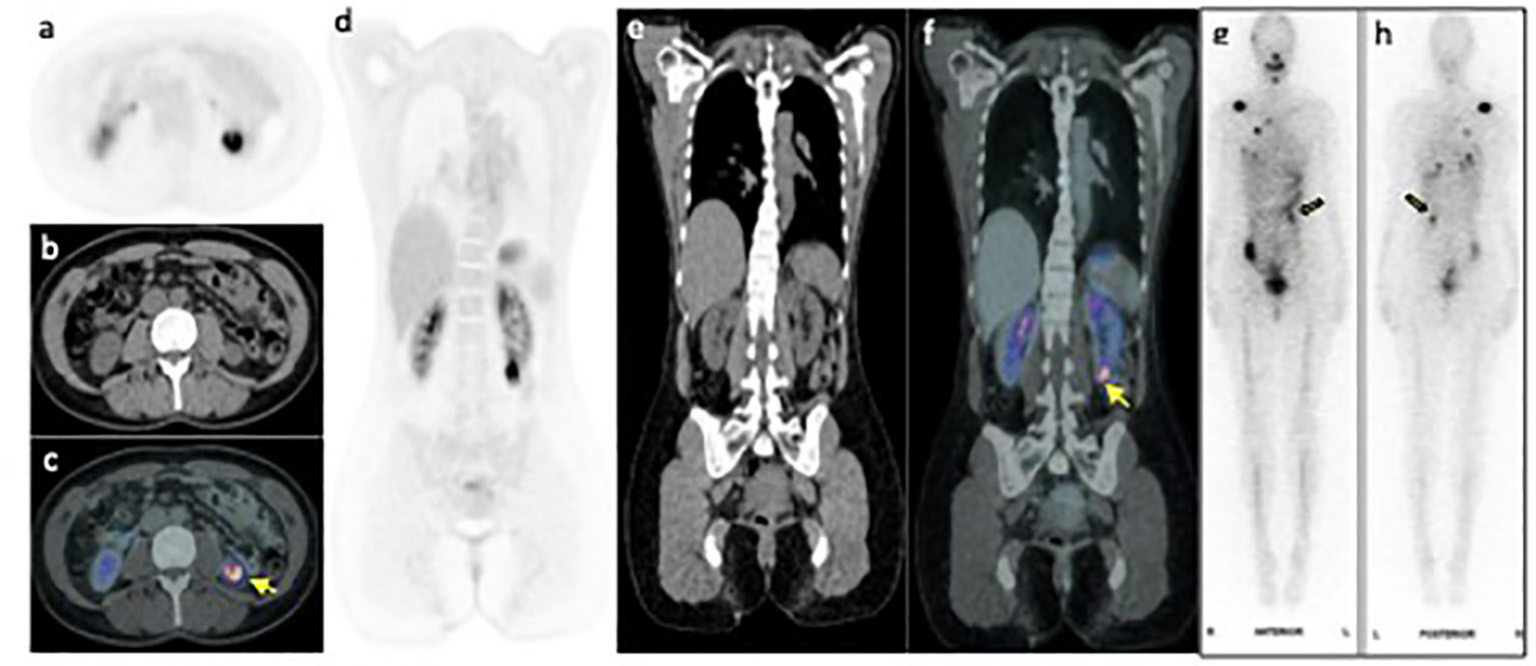
FDG PET/CT axial **(A-C)** and coronal **(D-F)** PET, CT and fused PET/CT images of the left lower pole renal mass (arrow) demonstrating intense FDG uptake with SUV max 11.5. Whole body I^131^ post therapy anterior and posterior scan **(G, H)** performed within 6 weeks of the PET image, shows I^131^ accumulation within the renal mass (yellow arrow).

**Figure 3:**
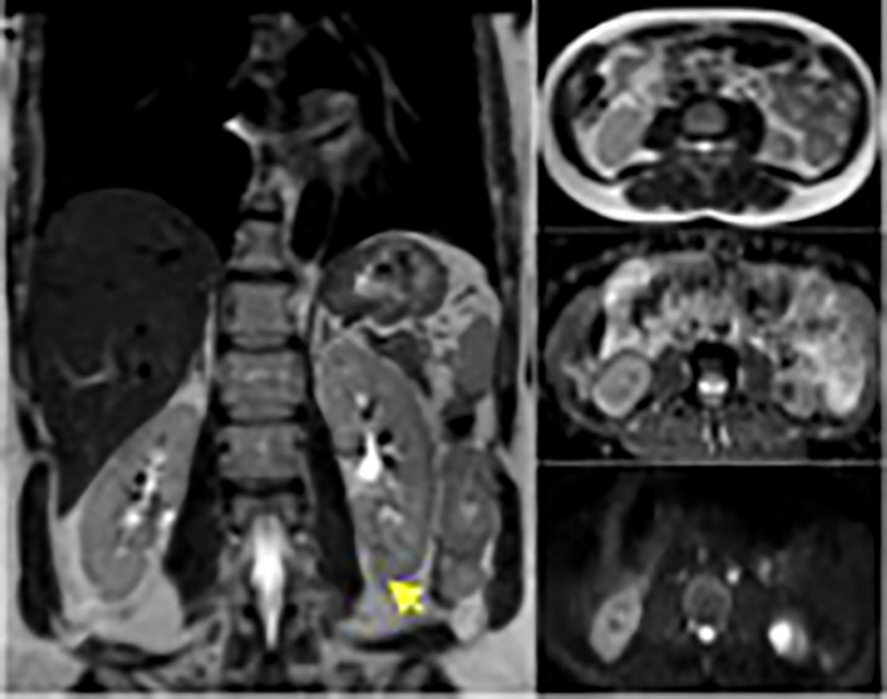
Enhanced MRI for the abdomen revealed solid enhancing left lower pole lesion with diffusion restriction.

**Figure 4:**
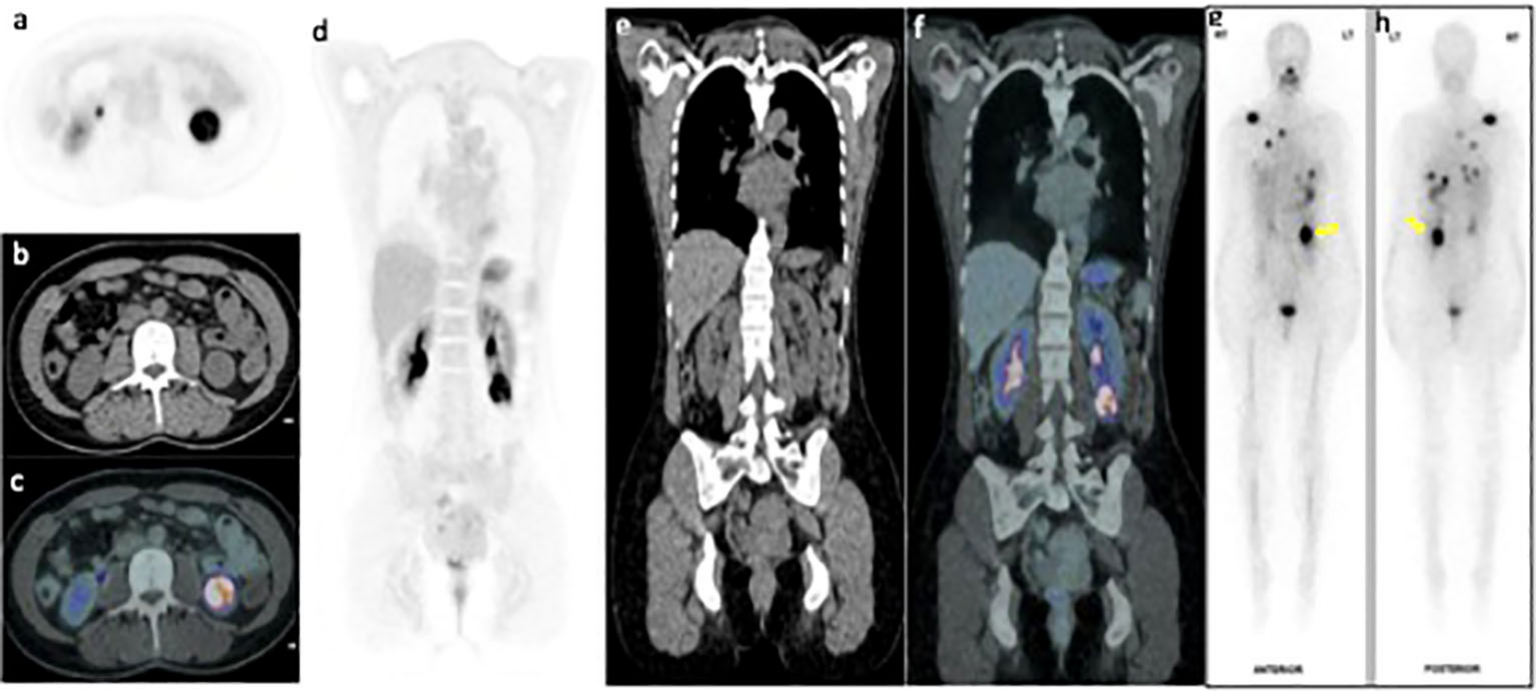
FDG PET/CT axial **(A-C)** and coronal **(D-F)** PET, CT and fused PET/CT images of the left lower pole renal mass (arrow) demonstrating interval increase in size and FDG uptake with SUVmax16.9. Whole body I^131^ post therapy anterior and posterior scan **(G, H)** performed within 6 weeks of the PET image, shows significant increase in I^131^ accumulation within the renal mass (yellow arrow).

The FDG PET/CT showed an incidental finding of benign non FDG avid renal mass consistent with renal oncocytoma based on morphological appearance (**Figure 1**). The renal mass demonstrates no iodin uptake in post therapy images. Over a follow-up period of two-years, this lesion showed interval increase in FDG uptake to a marked level (above reference liver) with yet minimal increase in size (**Figure 2**), as well as interval development of I131 uptake in consecutives post therapy images. The maximum standardized uptake value (SUV max) of the mass normalized for body weight was 11.5 and it measured 2.8 x 2.1cm. Enhanced abdomen MRI was performed, and it revealed a solid enhancing left lower pole lesion with diffusion restriction; this appearance was suspicious for a small renal cell carcinoma RCC (**Figure 3**). A 3-months follow-up FDG PET/CT (**Figure 4**) showed further increase in metabolic activity and size [SUV max 16.9, size 3.1 x 3.2 cm]. The interval increase in metabolic and morphologic features were not in support of renal oncocytoma as portrayed in the literature. This has hence raised the suspicion of well-differentiated RCC. Although metastatic renal disease is a possibility, the slow progress made it seem less likely. This warranted further histopathology evaluation which yielded a pathologic diagnosis of oncocytoma rather than well-differentiated renal cell carcinoma or metastatic renal disease.

## Discussion

Renal oncocytoma is usually asymptomatic and is observed incidentally during routine examination for non-urological abnormalities (1). It usually appears as a solitary tumor measuring between 4–8 cm, which may infiltrate peripheral renal tissues (2, 3). CT can reveal the presence of a solid homogeneous lesion with a centrally located scar, and arteriography may reveal a spoke-wheel vascular pattern (1, 4). However, these markers do not definitively distinguish oncocytoma from other renal tumors. As a result, numerous patients with oncocytoma are treated aggressively, due to the possibility of renal malignancy.

In the present case, the tumor was observed during routine examinations. Over a three-year of follow-up, there was interval increase in metabolic activity, although no evidence of local invasion or distant metastasis was demonstrated.

To our knowledge, there has been no published cases demonstrating a metabolic evolution of renal oncocytoma over three-year period, or renal oncocytoma of high I-131 accumulation on post-therapy scan.

Multiple studies have shown a relatively low sensitivity (31–77%) and high specificity (100%) for detection of renal masses with 18F-FDG PET (5–8).

Given the significant differences in management approaches of oncocytomas compared to RCC and other renal tumors, it is important to recognize that oncocytomas can yield false positive results on 18F-FDG PET, and absolute radiologic differentiation of oncocytoma from RCC remains challenging.

## Conclusion

This case study presents a rare evolution of renal oncocytoma in a patient with a history of papillary thyroid cancer. The tumor developed GLUT I transporter for FDG and sodium-iodide symporter expression, with accumulating I-131 over three years, highlighting the complexity and diagnostic challenges in such cases.

## Data availability statement

The original contributions presented in the study are included in the article/supplementary material. Further inquiries can be directed to the corresponding author.

## Ethics statement

Written informed consent was obtained from the individual(s) for the publication of any potentially identifiable images or data included in this article.

## Author contributions

AA: Writing – original draft. NE: Writing – review & editing. JB: Supervision, Writing – review & editing.
